# Mobilisation Alarm Use in Hospitals and Alignment With Person‐Centred Practice: A Qualitative Study

**DOI:** 10.1111/jan.70113

**Published:** 2025-08-04

**Authors:** Kelly Stephen, Dai Pu, Jennifer Weller‐Newton, Terry P. Haines

**Affiliations:** ^1^ School of Primary and Allied Health Care Faculty of Medicine, Nursing and Health Sciences, Monash University Frankston Australia; ^2^ Eastern Health Box Hill Victoria Australia; ^3^ Faculty of Medicine and Health Sciences, The University of Melbourne Melbourne Victoria Australia; ^4^ School of Nursing & Midwifery Faculty of Health, University of Canberra Canberra Australian Capital Territory Australia; ^5^ Rehabilitation, Ageing and Independent Living (RAIL) Research Centre School of Primary and Allied Health Care, Monash University Frankston Australia; ^6^ National Centre for Healthy Ageing, Monash University Frankston Victoria Australia

**Keywords:** alarms, attitudes, falls prevention, hospital, nurses, patient‐centred care

## Abstract

**Aim:**

To explore why and how staff use alarms for falls prevention in hospital and their alignment to person‐centred practice.

**Design:**

Qualitative interpretive design.

**Methods:**

One hundred focus groups and 25 interviews across 10 health services were completed between October 2022 and September 2024. Participants included nurses (*n* = 451), allied health (*n* = 82), and fall prevention managers (*n* = 18). The Framework Method guided initial data familiarisation and analysis and led to the Person‐Centred Practice Framework being identified as a useful framework.

**Results:**

Themes generated: (1) Understaffed, under‐resourced, under pressure, (2) Alarm impact on stress and workload, (3) Negotiating patient safety and patient preference, (4) Engaging family as a resource, (5) Sharing responsibility for alarms and falls prevention, and (6) Navigating ambiguity and fearing consequences.

**Conclusion:**

Staff feel compelled to use alarms despite problems associated with their use and challenges to person‐centred practice. Drivers of alarm use were feeling under‐resourced and fearing liability if patients fell. Staff want clearer organisational guidance in alarm use but also want the freedom to use their own clinical reasoning.

**Impact:**

Hospitals worldwide are working to identify effective strategies for preventing falls. However, research has yet to adequately explore the perspectives of frontline nurses and allied health staff regarding the use of mobilisation alarms—a critical gap when evaluating their impact and effectiveness. This study's six key themes provide insights into why alarms are so widely used despite the limited evidence supporting their effectiveness.

**Reporting Method:**

Consolidated Criteria for Reporting Qualitative Research.

**Patient or Public Contribution:**

This study did not include patient or public involvement in its design, conduct, or reporting.

**Trial and Protocol Registration:**

Australian New Zealand Clinical Trials Registry ACTRN12621000823875.

**Summary**

- What already is known?
  ○ Person‐centred practise is considered the global gold standard, though it can be challenging in busy, task‐oriented hospital environments.
  ○ Mobilisation alarms are routinely used in hospitals to prevent falls.
  ○ Robust evidence supporting the use of mobilisation alarms in hospital falls prevention is lacking.
- What this paper adds?
  ○ Staff often use alarms for falls prevention in hospitals due to feeling under‐resourced. However, alarms are less effective because of the staffing resources required to source, set up, and respond to them.
  ○ Alarms are used out of fear of liability and to avoid the emotional trauma of a patient falling under staff care. Yet in practice, alarms add emotional distress when staff hear them but are unable to respond promptly.
  ○ High alarm usage appears misaligned with person‐centred practice principles, which emphasise respect for individuals and their right to self‐determination.
- Implications for practice/policy
  ○ Adopting a more person‐centred falls prevention approach may reduce overall alarm use, or promote more individualised application of alarms.
  ○ Hospital leaders/managers could consider removing alarms as an option on management plans linked to fall risk assessment tools, given staff's tendency to interpret this as a mandatory strategy.
  ○ Fostering organisational cultures that emphasise steadfast support for staff, rather than apportioning blame, is essential.

## 1 Introduction

Person‐centred practice is the global gold standard in hospital care. It is a holistic approach involving healthcare workers partnering with patients and families to identify and address patients' needs, values, and preferences. Principles of individuality, self‐determination, engagement, compassion, and respect are critical for effective person‐centred practice (McCance and McCormack 2021). Systematic reviews have demonstrated an abundance of evidence supporting favourable effects of person‐centred practice for patients, families, and staff (Janerka et al. 2023). Nevertheless, a discordance exists between the person‐centred approach staff ideally would like to adopt and the reality of care they provide in busy, task‐oriented ward environments (Prato et al. 2019).

One area of practice that potentially challenges the concept of person‐centred practice is the use of mobilisation alarms for the prevention of falls. Theoretically, they are used to prevent falls by alerting staff when patients, who require assistance with mobility, attempt to exit their bed or chair without assistance. Falls are a common adverse event in acute hospitals, creating a clinical and financial burden (Dykes et al. 2023). Falls can result in psychological injuries such as loss of confidence, shame, and fear of falling in both patients (Kerr et al. 2023) and the staff responsible for patients who fall (Fehlberg et al. 2020).

## 2 Background

Globally, hospital falls remain problematic. Due to the significant harms associated with falls, substantial resources are spent on falls prevention. However, many interventions have proven ineffective. A meta‐analysis of hospital fall prevention interventions concluded alarms, when used as a single intervention approach, were not associated with significantly reduced falls (Morris et al. 2022). The World fall prevention guidelines reinforce this lack of robust evidence supporting alarm use and highlight their expense (Montero‐Odasso et al. 2022). There is the cost of the alarm systems themselves, as well as indirect costs–set‐up, inventory monitoring and repair, and the cost of staff time responding to false alarms, which were estimated to be as high as 52% of total alarm alerts (Brusco et al. 2021). All this staff time could potentially be better redirected elsewhere. In Australian health services, alarms were estimated to consume 11% of all falls prevention resources (Mitchell et al. 2018).

Patient and staff attitudes towards alarms are mixed. Some patients find alarms annoying, offensive, restrictive, and unnecessary, whilst others find them useful and reassuring of good care (Stephen and Campbell 2023). Staff grievances include alarms adding to busy workloads, they frequently malfunction, alarm noise increases patient agitation, high false alarm rates (Barker et al. 2017), concerns that patients who are not using alarms may miss out on care because of time spent answering alarms (Hubbartt et al. 2011), and alarms being considered a behavioural restraint (Okumoto et al. 2020). Given these concerns, one could question why alarm use persists and whether it aligns with person‐centred practice. Without a thorough understanding of the drivers of alarm use and the impact on staff and patients, there is a risk that alarms will continue to be relied upon in high numbers, placing additional stress on finite hospital budgets and the ability to provide person‐centred care.

## 3 The Study

This study explored whether person‐centred principles are applied to: (1) why hospital staff use alarms and (2) how hospital staff use alarms.

## 4 Methods

### 4.1 Design

A qualitative interpretive design (Thorne 2016) was chosen as it explores how people construct meaning in real‐world contexts, acknowledging the existence of multiple constructed realities. This approach aligned with the complexity and subjectivity of alarm experiences. An interpretative lens positioned the researchers' backgrounds as sources of insight, rather than biases to be eliminated. It enabled us to draw on our practical, theoretical, and research knowledge of alarms to inform data collection and analysis. The Consolidated Criteria for Reporting Qualitative Research (COREQ) was used to ensure reporting clarity and completeness (Supporting Information: File S1).

This research was undertaken embedded within two related multi‐site trials. First, a stepped‐wedge randomised disinvestment trial which investigated the effectiveness of alarms via gradual removal or reduction of alarms across 18 wards (Haines et al. 2021) and second, a quasi‐experimental study comparing the uptake of research evidence in the 18 disinvestment wards with another 18 wards who were not requested to remove or reduce alarms (Pu et al. 2024). The overarching trial results regarding alarm effectiveness and the impact of research participation will be presented in separate publications. This is the primary analysis of data in terms of addressing the research aims specified at the end of the background section. The issue of person‐centred practice was a key emergent theme from this analysis. Data collection did not set out to specifically explore this issue but emerged as participants shared their existing alarm practices, preferences, and practical challenges that led to high alarm rates. This knowledge supplements the quantitative results derived from the disinvestment trial (waiting publication) to form a more strategic communication and implementation approach should the disinvestment trial recommend disinvestment from high alarm usage in routine hospital care.

### 4.2 Study Setting and Recruitment

A purposive sample of 36 wards across 10 health services participated in the overarching research trials and also formed the data set for this qualitative study. Eight health services were from metropolitan Melbourne in Victoria, and two were from regional Victoria and New South Wales, Australia. Wards were a mixture of acute (*n* = 23), subacute (*n* = 12) or both (*n* = 1). All study wards used a high rate of alarms (defined as more than 3% of patients actively managed with alarms). Both ward staff (nursing and allied health) and fall prevention managers were interviewed. Recruitment was via convenience (ward staff) and purposive (falls manager) sampling according to the overarching research trials' timeline, which consisted of three time points that began in October 2022 and continued to September 2024.

### 4.3 Data Collection

Time point one explored staff's existing alarm practices and their reactions to the planned disinvestment research. It was anticipated that the prospect of future disinvestment may prompt deeper reflections on why and how alarms are used. Time point two explored any concerns about imminent alarm removal/reduction as part of the disinvestment phase and time point three explored staff reflections on participating in the research. Data relevant to the research question primarily emerged at time point one. However, participants not previously interviewed at time point one were keen to share their perspectives on routine high alarm usage at subsequent time points. Therefore, additional data continued to emerge revealing new themes and more in‐depth exploration of themes in the subsequent time points and were included in the present study.

Interviews occurred on the wards or online. Staff working on the day of the visits were invited to be interviewed and those who accepted gave informed consent. No participants withdrew consent. One hundred ward‐based focus groups (*n* = 100) and interviews (*n* = 25) with fall prevention managers were conducted. Ward focus groups averaged 20 min and fall prevention manager interviews averaged 29 min.

A semi‐structured interview guide developed by the research team was modified as needed to explore concepts encountered in earlier interviews. No radical changes in the interview guide were required. Interviews focused on the following areas: feelings about the evidence regarding alarm effectiveness, feelings about alarm reduction or removal, training and clinical reasoning about alarms, alarm response and how alarms might be more effective (Supporting Information: File S2).

Interviews/focus groups were facilitated by KS (a female PhD student and occupational therapist) and DP (a female academic researcher and speech pathologist), both with qualitative research experience. KS was employed at one participating health service but had no involvement in ward activities. Participants were aware that the researchers completing the interviews were also coordinating the disinvestment trial.

Interviews/focus groups were audio recorded, auto‐transcribed via Microsoft 365, and manually reviewed for accuracy and completeness by KS. Interview transcript summaries were emailed to participants, and they were invited to provide feedback. Three participants requested minor amendments, which were adopted. Reflexive discussions between researchers and recording of field notes occurred immediately post interviews/focus groups.

### 4.4 Data Analysis

Transcripts were uploaded into NVivo version 14 (Lumivero 2023). The Framework Method (Ritchie et al. 2003) was chosen as an analytic tool. The Framework Method facilitates systematic categorisation and organisation of large data sets. The five steps as described by Ritchie et al. (2003) were followed. First, data familiarisation occurred via transcription and summarisation of participants' transcripts for member checking. Transcripts were annotated as they were uploaded into NVivo for key ideas. Second, via reflection of the annotations, it was evident that variations in person‐centred practice featured in how alarms were used. Hence, the Person‐Centred Practice Framework (PCPF) (McCance and McCormack 2021) was identified as a useful framework to deductively code data.

The PCPF is a theoretical model which can guide clinical practice, leadership, learning, research, and quality improvement. Its five key domains articulate the complex and dynamic nature of person‐centred practice (PCP) at different levels within healthcare systems. The domains are ordered so that the Prerequisites (Domain 1—staff attributes) and The Practice Environment (Domain 2—the context) must be effectively managed for Person‐Centred Processes (Domain 3) and Outcomes (Domain 4) to be achieved. These domains are embedded within the broader Macro Context (Domain 5), which is strategic and political in nature.

The third stage of the Framework Method involved indexing the PCPF systematically to all data. The Prerequisites and The Practice Environment domains were chosen to code data against given they are the building blocks of PCP. The fourth stage involved the creation of separate matrices for Prerequisite and Practice Environment domains to summarise and analyse the data in totality. The final stage involved an inductive process of mapping and interpretation where patterns of attitudes towards alarms were explored in the matrices. Themes and sub‐themes were generated by KS and further revised with input from the research team.

### 4.5 Ethical Considerations

The overarching alarm disinvestment trial was registered with the Australian New Zealand Clinical Trials Registry: ACTRN12621000823875. Ethics approval for qualitative data collection was granted in late 2021 within the application of the overarching disinvestment trial by the Monash Health Human Research Ethics Committee (RES‐21‐0000‐468A). Site‐specific approvals were obtained from all participating health services, and individual verbal and written consent was obtained from interview/focus group participants.

### 4.6 Rigour and Reflexivity

The researchers were aware of the uncertain evidence base supporting alarm effectiveness. The disinvestment trial's objective was to produce evidence examining the effectiveness of alarms to prevent falls, whilst this qualitative study sought simply to understand the justifications for why and how alarms are used. The researchers' personal clinical experience of occasions when alarms were and were not useful helped them to build rapport with participants and ask insightful questions to probe deeper into both positive and negative responses about alarms. Care was taken to ensure no leading questions were asked and the researchers emphasised there were no right or wrong responses.

Participating health service managers were motivated to nominate both wards led by nursing managers who were keen to explore alarm disinvestment as well as opponents who valued alarms, to encourage diverse perspectives. All available staff across disciplines were invited to participate in interviews, and multiple visits were offered to capture a breadth of staff experiences.

Qualitative research experience within the research team and backgrounds from nursing, physiotherapy, speech pathology, and occupational therapy reflected the participants' disciplines and permitted interpretation of data from diverse perspectives, reducing the risk of individual biases. All interview transcripts were analysed to ensure any unexpected findings were not overlooked. High‐level summaries of themes were verbally shared with participants to provide opportunities to challenge our interpretations.

## 5 Findings

### 5.1 Participants

Participants consisted of Nurse Unit Managers (*n* = 33), ward nurse leaders (*n* = 83), nurses (*n* = 335) and allied health (*n* = 82) see Table 1. Fall prevention managers had a combination of nursing (*n* = 14) and allied health (*n* = 4) backgrounds. Whilst fall prevention managers were often consistent across time points, most ward staff were only available at one time point due to the lengthy periods between data collection points, staff turnover, and shifts worked. Fifty‐eight staff (10.5% of total participants) were interviewed across multiple time points.

**TABLE 1:** Participants.

Health service	Hospital ward	Ward type	Nurse unit manager			Nurse leader			Nurse^a^			Allied health			Fall prevention manager (KI)		
			T1	T2	T3	T1	T2	T3	T1	T2	T3	T1	T2	T3	T1	T2	T3
A	A1	GEM/ACOP	1		1	3	1	1	2	3	8	2	2	1			
	A2	Acute renal			1	1	2	1	9	4	11			2			
	A‐KI														1	2	1
B	B1	Acute ACOP	1	1		1	5	3		10	8		5	4			
	B2	Acute renal		1	1	1	5			6	5						
	B3	Acute medical				1		3	7		5	1					
	B4	Acute medical						1			8						
	B‐KI														1	1	1
C	C1	Acute med/oncology	1		1	2		1	1		6						
	C2	Rehabilitation	1		1	1	1	1	4	3	10	6					
	C3	Acute medical	1		1	3		1		5	11	1		2			
	C4	Acute med/oncology	1		1				5		3	2					
	C5	Acute medical	1		1	2	2	3	10	5	3						
	C6	Acute neurology	1	1	1	1	1		3	8	5						
	C7	Acute neurology	1		1		2		4	10	6						
	C8	Acute orthopaedic			1			1			2						
	C9	Acute medical			1						1						
	C‐KI															1	1
D	D1	Rehabilitation/GEM		1	1	1	4	1	4	5	7	8	1				
	D2	Acute medical	1			2	2	1		3			1	9			
	D3	GEM	1				1		5			4					
	D4	Neurological rehabilitation/GEM			1			4			4			5			
	D5	Acute neurology				1		1	1		6						
	D‐KI														1	2	1
E	E1	Acute medical	1				2		10	6	6					1	
	E2	GEM		1	1	1	1	2		2	6		4	2			
	E3	Rehabilitation		1	1	1	1	3	5	4	5		3	1			
	E‐KI														1	2	3
F	F1/F2	Acute/Rehabilitation			2						4			1			
	F‐KI																2
G	G1	Acute	1					1	2								
	G2	Rehabilitation			1			2						2			
	G‐ KI														1		4
I	I1	Acute medical			1				8		1	1		1			
	I2	Rehabilitation/GEM	1						7		1	4		1			
	I‐ KI														1		1
J	J1	GEM						3			8	5					
	J2	Acute surgical	1		1	3		1	6		9			1			
	J3	Acute medical				1		1	2		5			1			
	J4	Acute medical				1			5		8			1			
	J5	Acute neurology				2		2	2		7			1			
	J‐ KI														1		1
K	K1	Transition Care			1						1			2			
	K2	GEM			1						3			2			
	K‐ KI														1		1
**Total number**	**10 health services** **36 wards**	**23 Acute** **12 Subacute** **1 Acute/subacute**	**33 Nurse Unit Managers (11 interviewed across multiple time points)**			**83 Ward nurse leaders (12 interviewed across multiple time points)**			**335 nurses (18 interviewed across multiple time points)**			**82 allied health (7 interviewed across multiple time points)**			**18 Fall prevention managers (10 interviewed across multiple time points)**		

*Note:* T1 – Time point 1 (baseline), T2 – Time point 2 (one week prior to disinvestment commencing), T3 – Time point 3 (following disinvestment). Nurse leader included associate nurse manager, clinical nurse specialist or clinical nurse educator. KI – Key informant included falls prevention manager or staff member involved in organisational falls governance. Allied health—included occupational therapist, physiotherapist, speech therapist, allied health assistant, diversional therapist, porter.

Abbreviations: ACOP, acute care of older person; GEM, Geriatric Evaluation Management.

^a^ Nurse included student nurse.

The participants' attitudes were viewed through a PCPF lens. KS developed alarm codes specific to the PCPF's Prerequisite and Practice Environment domains and tested them using data from a subset of transcripts. Following agreement of the research team, the remaining transcripts were deductively coded by KS using the alarm codes. New codes were inductively added when new data did not align with existing codes. The final code structure is illustrated in Table 2.

**TABLE 2:** Alarm codes in relation to PCPF Prerequisite and The Practice Environment domains.

Domains			Codes				
Prerequisites definition	Clarity of beliefs and values	Commitment to the job	Developed interpersonal skills	Knowing ‘self’	Professionally competent		
*Staff attributes to deliver effective person‐centred care and manage fall prevention challenges in constantly changing hospital environments*	Valuing shared decision making Valuing person‐centred care Congruence between values and alarm use	Dedication to providing the best care How alarms are used/answered Exploration of the most appropriate fall prevention interventions Influence of leadership	Verbal/nonverbal observations of impact of alarms Patient/family consultation about alarms Knowledge of patient routines	Self‐reflection of how alarms are used Stress level and awareness Openness to feedback in approach to falls prevention/alarms	Knowledge of fall risk/fall prevention interventions Experience using alarms Knowledge reaction to evidence/research participation investigating alarms		

Practice environment definition	Staffing and appropriate skill mix	Effective staff relationships	Physical environment	Potential for innovation & risk taking	Power sharing	Shared decision making	Supportive organisational systems
*Regardless of the attributes of staff, unless the care environment is conducive to person‐centred ways of working, the true potential of working in teams to prevent falls cannot be fully achieved*	Experience levels of staff and impact on alarm use Influence of staff ratios	Support for each other in alarm decisions Support for each other in responding to alarm alerts Support for each other more broadly in fall prevention	Access to high visibility or shared rooms Access to other fall prevention devices Alarm device type including sensitivity and maintenance requirements	Sharing information to make informed decisions about alarms and falls prevention Evidence based practice	Hierarchical relationships between patients and staff Hierarchical relationships among staff Role clarity between nursing, allied health and medical Appreciation of different team members	Processes facilitating shared decision making about alarms Processes facilitating shared decision making regarding research involvement investigating alarms	Promotion of initiative and safety Liability concerns if patients fall Fall prevention or alarm policies/guidelines

**FIGURE 1:**
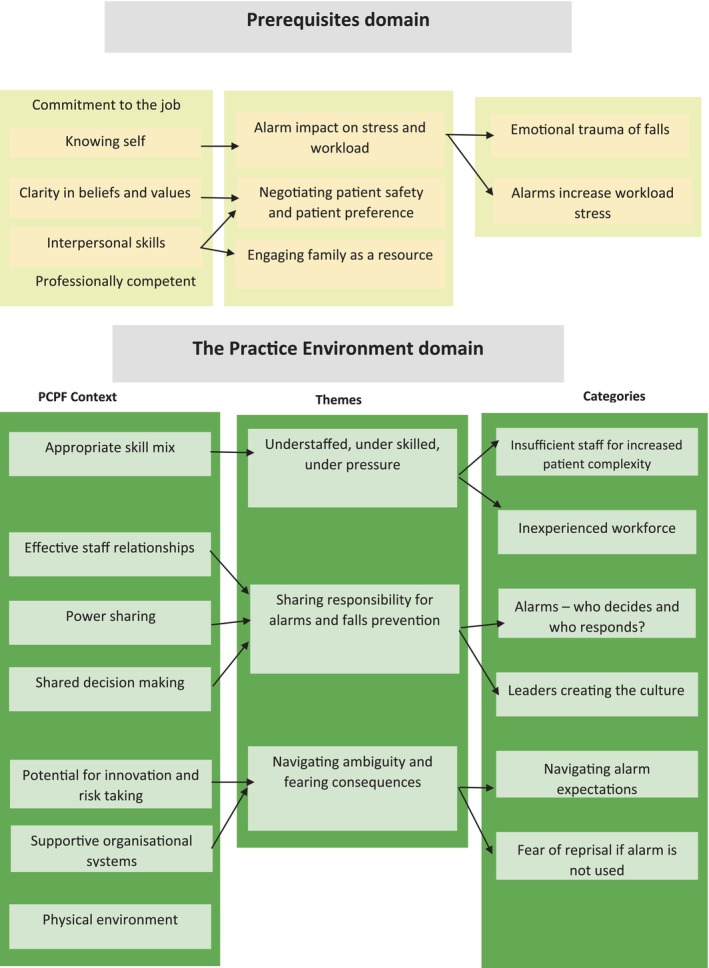
Themes mapped to PCPF prerequisite and the practice environment domains.

The six themes generated related to why and how staff use alarms and were connected to explanations as to why alarms may not prevent falls and challenged PCP: (1) Understaffed, under skilled, under pressure, (2) Alarm impact on stress and workload, (3) Negotiating patient safety and patient preference, (4) Engaging family as a resource, (5) Sharing responsibility for alarms and falls prevention, and (6) Navigating ambiguity and fearing consequences. Figure 1 provides an overview of themes mapped to the PCPF. Table 3 provides numbered quotes referenced in brackets under each theme to provide evidence for the researchers' interpretations and provide the participants with a voice within the study.

**TABLE 3:** Participant quotes.

Quote #	Theme	Ward/manager
	**4.1 Understaffed, under skilled, under pressure**	
1	For that period from admission overnight when they’re more confused or they’ve come from emergency and they’re over stimulated and there's all of those things, you just don’t have the staffing to be more available. That's when you probably throw an alarm on.	B1
2	Our alarm is not being used properly or effectively because of lack of resources.	A1‐AH
3	I don’t think there's a lot of time to, or time and space to, think about the clinical reasoning providing an alarm.	C3/4 AH
4	Say the patient has just come in, you don’t know their mobility status, you’re waiting for physio, you’re waiting for OT, you’re waiting for them to be assessed. You put them on the sensor as a precaution.	G1
5	My thing is on night duty, when we can’t kind of look at every patient, we have got 9 or 10 patients. What if they are on the floor for two hours and we don’t know?	B3
6	If we’re specialing a patient one to one, the way we would extend that out is perhaps by using a bed arm … so it's 1:00 to 1:00 and then we maybe go 2 nurses for eight patients but with a bed alarm … we’re told to run staffing pretty tight.	C7
7	There's a lot of new grads with not enough clinical supervision sometimes, a lot of new staff just rotating and chopping and changing … I don’t think there's a lot of time and space to think about the clinical reasoning providing an alarm.	C3/4 AH
8	I am approaching and I am responding, but … new staff, they are not even realising that they are not even sensing them straight away and not approaching straight away. Majority of the working group nowadays, not having like me, having that feeling ‘oh yeah the sensor is going ‐ have to (respond).’ Our staff in here, new inexperienced staff.	E2
9	We’ve got reduced skill mix on wards, we have much higher acuity on the wards … they do need a lot of support and education to grow and really develop that critical thinking.	K‐K.I
	**4.2 Alarm impact on stress and workload**	
10	Emotion can be really elevated. It can have a big impact on people in how they perceive themselves within their career.	E1
11	I get a lot of staff concerns competing and I want to reassure them always and be like, ‘well, you can always use the bed alarm’	B2
12	Sometimes when we’re in a stressed environment … those (proactive fall prevention) strategies fall by the wayside.	B1
13	I’m worried (without alarms) people won’t go on their break because they are worried about their patient load, it might affect morale by not getting breaks because you’re nervous, or anxious, worried about what can happen … the alarm is a safety net.	B3
14	When I’ve got a patient on a Proxi it gives me more confidence. Like that I can come to the Med room and take my time. I don’t have to constantly be checking them.	J3
15	I find it really frustrating that that's (alarm/CPO) all that people throw at the falls.	D2
16	Patients just kind of moving, it's kind of triggered it, even like breathing, the patient has stayed still. It's just like ‘I’ve still got things to do and this is annoying.’	J3
17	If even one of them is on a bed sensor, you don’t have time to scratch yourself, let alone do any proper care for anybody.	E1
18	You get handed over the patient's got a proximate alarm. So you think, OK, they’ve got a Proximate, you might not go directly to that person. And then when you go there, you find it's not functioning properly. It's not placed in the right way or whatever. So, you have to be double vigilant to what you were if you’re just using your own common sense and knowledge and documentation, than having to wonder if something is working not working. … I would rather rely on this (points to head), than think there is a safety net and then it's actually not a safety.	D2
	**4.3 Negotiating patient safety and patient preference**	
19	There is often the stupidity, where it is insisted that we keep the chair alarms on patients who are turning them off and you think ‘well, why?’ They’re turning them off! If they’ve got the cognition to turn them off.	B1‐NUM
20	I’ve got a patient at the moment who has had the alarm and she refers to it as the ‘dobber.’ Like she's very anti the alarm … It dobs on her when she moves.	C2
21	Patients tend to get more frustrated if they think an alarms going off, as if we’re kind of helicoptering and managing them, as opposed to, they might not necessarily know that the type of rounding that they get is increased. They might think, “oh, everyone's quite attentive here.”	C‐AH
22	If you’ve got a delirious patient or a patient who might be potentially aggressive, it can sometimes set them off … and everybody else in the room	J3
23	They are usually just telling them to shut up (laughter, murmur of agreement). ‘What's that noise? What have I done?’ And it's like, ‘oh, yeah, just thought I’d come and see you’.	C3
24	They don’t want the alarm, but we want some mechanism to identify they’re actually getting up because our problem is they’re so weak or fatigued or their balance is an issue … they can end up falling. So the question you’ve got to raise is: How do we manage the safety factors in the best possible way with a patient who may not be compliant for what they need?	C4
25	A lot of the patients don’t like it at all and they will do everything to avoid that mat. And once they know what that's for … it just gets dangerous and we have to remove it anyway. As I say, we have people jumping over	I2
26	You can’t just give it to them … I try to explain to them … he knew what it was for, he didn’t like us using it, but towards the end he was accepting of us using it for safety for him.	G1
27	The patients aren’t happy with it, there are no patients happy with an alarm, they are always like ‘what's this one?’ They’re going to remove it … We just trick them, we put it under the sheet.	J3
28	(Use alarms so) the patients who like to be naughty can’t get away with it. … That's a truthful way of putting it.	E3
	**4.4 Engaging family as a resource**	
29	The reality is, we don’t have enough staff on the ground, we can’t fill shifts and it actually comes back to having trained people on the floor, anticipating those patients’ needs or their family members being informed	B‐K.I
30	The families definitely like them. The first thing family would say if somebody had a fall was "didn’t they have an alarm on?"	K2‐NUM
31	Family misconceptions of alarms “like a medication … you took the antibiotic it has helped with infection, same with the falls alarm, you took the magic thing and it works.”	A1
32	Some patients are regulars … ‘Is mum on a hi‐lo bed, are there mats on the floor next to her? The socks on?’ Like they’re kind of aware because they have been in hospital settings a lot, or ‘mum has had a nasty fall before, are these in place to prevent? … it is already pre‐asked before they are even on the ward sometimes.	B3
33	The only time we’ve had family involvement would be around they’ve come in when we’re doing an assessment, the alarms going off and we explained why that's in place. I haven’t really had too much feedback in terms of their thoughts around them.	A1‐AH
34	I talk to family that we don’t think it will be beneficial, but we give the family that option. We just kind of say, ‘we don’t think it will be that beneficial. We think it might irritate them more, but if you would like to trial the sensor we can have a look at it.’	E3‐AH
35	In my country … the family will stay the whole day, they actually help in terms of toileting, preventing falls, it would be effective, but here in Australia we can’t really mandate that …. in my country … we have less falls because the family is there and help in terms of their ADLs … the nurses just go and give medications, do obs or other nursing procedures. In terms of ADLs or toileting, the family is there helping. It is like security base.	G1
	**4.5 Sharing responsibility for alarms and fall prevention**	
36	In terms of us having a formal process for discussing with the patients, discussing with family when we’re putting them in or taking them away, we probably don’t have that covered off.	F‐K.I
37	There is no insight into their care. They don’t know why they are on a chair alarm, they don’t even know why they are in hospital.	B3
38	Everybody knows there is a bed alarm and where the bed alarms are. It would be really silly to disrespect that.	C8
39	Supposed to be everybody's responsibility, but it is not. (Laughter) It's everybody's responsibility, but the nurses have allocated patients so … (laughing)	D1
40	(Alarms are) not initially driven by Allied Health staff and we kind of more wonder, we’re not involved in the process of putting them on, so we kind of watch it and go ‘hmmm.’	I‐K.I
41	I don’t know if that's really in the physio's hands cause then that goes out of mobility and into actual behavioural … an impulsiveness to get up and go. That's why they’re on it. Maybe that's not actually the physios’ call because it's now off their mobility and to what we deem if they are safe or not.	B4
42	(Alarms decided by) the wider team, but we overrule.	D1
43	Often people think the solution is ‘we’ll put a chair alarm on them’. And I’m like, ‘no, not necessarily’. They’re not the ones answering those chair alarms, that's the whole thing … I’m not against allied health but that becomes their thing ‘oh, put a chair alarm on’ but you’re not answering them.	B1
44	‘This patient is high risk of falls. It's a recommendation is for falls alarm. That's it. I ticked my bit, I’ve signed and I’ve recommended, I told the nurse. That's fine, that's it.’	A1
45	I think once people get labeled as like impulsive and the alarm gets put on, there's no like weaning process or like "how are we gonna take this away?" And it's very much an allied health driven	B1‐AH
46	There's the prospect that falls risk prevention becomes the total domain of nursing staff. But it's not, it's Physio, pharmacy and Medical staff … and family … we just want to pigeonhole everything and make everything a nursing responsibility, which means that we have to find a solution.	J2
47	The alarm would be going off, and they’re (allied health) just sitting there, they’re at the desk doing nothing. If they come on board and actually helped out. … Every now and then we get a really good lot (allied health), but most of the time they just think ‘nothing to do with us’. … I mean, how hard is it really? Unless we get everyone else on board to help out, it doesn’t matter what we do, it won’t work. And that ain’t hard because they’re already here.	D1
48	We need to be coming up with patient‐centred solutions as a team … really work together to develop something, ‘cause it's what the national standard for comp care is about right? Everyone coming together to develop the one plan of care, personalized plan of care.	D‐KI
49	Within my role, it is about explaining to people the evidence. ‘The evidence in the guidelines, this is what they are saying….’ So it is putting the information out there in regards to what the evidence is, but it's also listening because they’re telling us clinically they want the alarms.	G‐K.I
50	Rather than relying on the Nurse Unit Managers to be the business owners for all pieces of equipment, they’re starting to see the benefit of centralising and putting that money to perhaps staffing or other more needed strategies.	B‐K.I
	**4.6 Navigating ambiguity and fearing consequences**	
51	They were brought in and then everybody had to have them. It was like, ‘this is going to solve all the falls we have in the organisation.’ They became mandatory, we had to use them.	J2‐NUM
52	People were just randomly taking patients off Proximate alarms and then they would fall again. So the rule was you weren’t allowed … so the decision to take a patient off a Proximate alarm needed to be NUM or aNUM decision.	J2
53	When the patient started on a bed alarm, it's hard for them to get rid of them. You feel like, ‘we’ll just continue until the patient goes’ because all about safety. So it's really hard to pull them back. …. We over protect them, we’re like ‘oh, we’ll just continue until the patient goes … because if someone removes the bed alarm and then the patient has a fall the next day, the first thing is gonna be like, ‘why was the bed alarm taken off?’	C6
54	They (physios) will say high falls risk. The minute you say high falls risk, you sort of box me into a corner that tells me I need to put in place interventions to protect the patient, or prevent risk of harm. But the discussion I have with them is, they go ‘oh, they don’t need a Proxi, but they are a high falls risk.’	J2‐NUM
55	At the end of the day, despite how many strategies we can put in place, it's my registration on the line. If it was to go to Coroners, or something like that, for those really, really bad cases, it makes me nervous about my registration and, how much the hospital will look after me … And if a bed alarm is something that could have been very easily implemented	C6
56	Sadly I’ve got to stages where I get staff to put the chair alarm on so when they do fall, because this patient is going to fall anyway, I don’t have to get the back flack that says ‘why didn’t you put that patient on a chair alarm?’ at in depth case reviews. …To be honest, it is to cover myself and to make my life slightly easier when I’m doing that in depth case review or doing the VHIMs to say ‘well we did our best’, even though I know it isn’t necessarily going to make any difference, but at least I’m trying to cover myself and my staff.	B1‐NUM
57	It also comes down to the management on the ward. They’re not necessarily gonna take that confidence from us if the culture is still ‘well, if you say don’t use one, but then I don’t and the patient has a fall, it’ll be my NUM on my back, not you’. … So we can definitely encourage (reducing alarms), which I sort of try and do anyway, but they (nurses) don’t report to us.	B1‐AH
58	I always advocate for the junior staff to treat the discussion as a shared decision making process, so really talking to the nurses about the 24 h behaviour … and how they’ve been managing … it's also unfair on the nursing staff to have to make that singular decision as well, when there is so much liability associated with it. So to be able to talk it out and make it a shared decision making process helps.	J‐AH
59	We see a real disconnect and almost like probably a compliance lead motivation to complete the fall screen form than anything that's meaningful for the patient.	F‐K.I‐1
60	It's (assessment form) stopping us from being nurses and looking at the patient and say ‘as a nurse this is what I think is happening with this, I think the patient needs it’ sort of makes us rely on a form to tell us that.	E3

### 5.2 Understaffed, Under Skilled, Under Pressure

#### 5.2.1 Insufficient Staff for Increased Patient Complexity

Bed alarms will pick up the slack if there's not enough staff (C6)

Participants reported patients were becoming increasingly complex and they felt inadequately staffed to meet workloads. Occasionally, alarms were used in substitution for proactive visual checks. Alarms were considered useful overnight due to reduced staffing compared to daytime ratios (1). Paradoxically, alarms were also believed to be ineffective because there were not enough staff to answer them promptly to prevent falls (2). Staff were often responsible for multiple alarms and became stressed when presented with the impossible task of responding to simultaneous alarms or leaving an important task or high fall risk patient to attend an alarm. Asking colleagues to help answer alerts also provoked stress because staff were aware everyone was busy.

Participants believed they lacked time to explore potentially more relevant fall prevention plans which identified and addressed why unsteady patients attempted to walk unsupervised (3). Alarms were a quick, easy, and widely accepted solution. Allied health was considered a valuable resource to assist in developing individualised plans; however, they did not work after hours (4).

Complex patients often required multiple nurses to assist with care for longer time periods, again limiting staff's ability to proactively check other patients. Many staff questioned the effectiveness of alarms in preventing falls. Yet they felt alarms were possibly effective in reducing harm by preventing complications from long lies where staff had been unaware a patient had fallen because they were stuck caring for other complex patients (5).

Ongoing use of Constant Observers was restricted due to fiscal constraints. Constant Observers (also known as ‘specials’ or ‘sitters’) are additional staff members, such as nursing assistants or security guards, who under the delegation of registered nurses provide increased observation of patients at risk of harm including falls. Alarms were considered a good step down from using more expensive Constant Observers (6).

#### 5.2.2 Inexperienced Workforce

The challenge of the soft skills we have with the more junior workforce … there's lots of opportunities for improvement (B‐K.I)

Most allied health and nurses had never experienced working without the option of alarms and busy clinical demands combined with lack of supervision meant alarms could be prescribed as a default strategy (7). Nurses expressed strong beliefs falls would increase if alarms were unavailable. However, older and more experienced nurses remembered when alarms were not part of usual care and, alongside allied health, felt more confident alarms were not essential. Some participants lacked confidence in their clinical reasoning and, even though they doubted the effectiveness of alarms, felt alarms were better than nothing. Junior staff were accused of lacking an understanding of the importance of falls prevention and the need to answer alarms promptly (8). There was frustration that not all staff practice at the same level despite attempts to standardise care. Some staff appeared unaware of how their interactions with patients, or the alarm itself, can inadvertently contribute to agitation and fall risk. Managers believed the Covid pandemic limited clinical placements and learning opportunities to develop critical thinking and led to staff defaulting to alarms as a first‐line strategy (9). Senior nurses, despite trying to avoid high alarm rates, defaulted to alarms when they needed to spend excess time onboarding new inexperienced staff.

### 5.3 Alarm Impact on Stress and Workload

#### 5.3.1 Emotional Trauma of Falls

If you've got a patient who has been deemed a high falls risk, then you are looking for a mat for that patient, you feel like a criminal if you can't find one, you think ‘they're going to fall and it is my fault’ (I2)

Nurses identified their primary role was to keep patients safe. Falls can impact how staff perceive themselves within their careers (10). They are petrified patients will fall, and want to implement all available strategies. In‐charge nurses advised staff to use alarms to relieve both theirs and their staff anxiety (11). Staff acknowledged in highly stressed wards alarms provided reassurance if some strategies were overlooked (12). Without alarms, there was a sense that staff would burn out with worry their patients would fall (13), and alarms offered a reprieve from constant vigilance (14).

#### 5.3.2 Alarms Increase Workload Stress

When I hear multiple alarms, I do get very ‘need to go to it. I need to go to it.’ But I can't. I have to do this and sometimes it will render you immobile … you get lost. It's like buzzers and tasks. And then you forget the patient (A2)

Alarms are part of routine practice and a source of comfort to nurses that this is how falls risk is managed on their ward. Potentially staff continued to use alarms to avoid the stress of change. Differing staff views on alarm usefulness also caused stress, as staff negotiated conflicts within the team of who should and should not use alarms, when they should cease and which staff were or were not responding (15). Staff were annoyed by frequent false alarms (16) and frustrated that alarms took away from other important tasks (17). They also worried if the alarm was turned on, positioned correctly, working properly or who was available to answer it. So, whilst some staff felt alarms saved vigilance, others thought alarms required them to be doubly vigilant (18).

### 5.4 Negotiating Patient Safety and Patient Preference

This disturbs sleep too. Always changing position and it's going off and patients ask ‘can you turn it off?’ And we say ‘no, it's for your safety.’ That's where I've found it difficult (B1)

The drive to keep patients safe appeared to come at the expense of person‐centred and shared decision making values. Staff consistently described an awareness patients did not like alarms via explicitly being told by patients or observing their behaviour, but they still felt compelled to use them (19). Researchers observed that staff invariably laughed when asked about patients' feelings towards alarms, potentially trying to downplay the significance of disregarding patient preference. Multiple reasons were given why and how patients experienced alarms negatively such as: feelings of restriction (20) or intrusiveness as staff repeatedly responded to alerts (21); excessive noise agitating both patients using alarms and their co‐patients (22); exacerbating confusion (23); prompting unsupervised mobility as patients searched for the source of the noise or attempted to turn off or avoid the alarm. All these alarm reactions can heighten fall risk as well as impact staff‐patient rapport. It is unclear how staff negotiated the risk of alarms triggering negative experiences with the potential benefit of preventing falls (24). Patient choice was often overridden. However, there were also instances where alarms were removed based on patient discomfort (25).

Including patients with cognitive impairment in alarm decisions was difficult due to these patients' reduced insight and inability to remember fall prevention strategies such as calling for assistance or using their gait aid. Consequently, alarms were often introduced without consent or patient consultation. Other times, more formalised discussions occurred (26). Staff occasionally resorted to covert practices such as hiding alarms (27) or using alarms like a punishment when patients walked unsupervised (28).

### 5.5 Engaging Family as a Resource

I had relatives swearing at me that we've let this happen and no alarm (B1)

Implementing alarms was quicker than proactively engaging family for more individualised interventions (29). Reasons staff prioritised alarms over proactive family engagement were time constraints, limited staff experience and confidence, lack of formal processes to establish who was responsible for family engagement and impracticality of family input overnight. Managers highlighted family engagement required improvement.

Alarms were used to reassure families (30). Some relatives believed alarms guaranteed patients would not fall (31). Many staff felt ill‐equipped to justify non‐use of alarms to families and so continued using them. Whilst families of frequently hospitalised patients occasionally proactively requested alarms (32), alarm decisions were usually made solely by staff and then retrospectively communicated to family (33). Examples of proactive family collaboration existed, though were less frequent (34).

Participants raised alarms were uncommon in some parts of Asia. Families were culturally expected to provide assistance with daily care in hospital. This allowed nurses to concentrate specifically on nursing duties, and they perceived family presence positively reduced falls (35).

### 5.6 Sharing Responsibility for Alarms and Falls Prevention

#### 5.6.1 Alarms—Who Decides and Who Responds?

The patient had turned the mat off themselves because it was annoying them. If they've got the insight to do that, they probably could be part of a good plan … they could be at the centre of their own care plan if they have got the capacity (I‐K.I)

Staff reported no formal procedures around who implements or removes alarms. Practice varied among wards. Staff agreed it was a staff, rather than patient‐led decision (36). Patients' cognitive impairment was cited as a barrier to their involvement (37). Often the bedside nurse organised alarms, or doctors and allied health handed over alarm recommendations for nurses to implement, but with no accompanying discussions regarding patient/family collaboration.

Responsibility for alarm decisions and responding to alerts varied from an individual nurse to a team's responsibility. Some nurses described themselves as great communicators and problem solvers—always discussing and managing fall risk. In these cases, the usefulness of alarms was reportedly reviewed regularly, alarms were not overused, alarm responses were prioritised by all, and alarm pagers were left at nursing stations with expectations that colleagues would respond, rather than out of negligence (38). More commonly, staff did not prioritise answering alarms outside their assigned sections (39), thus increasing response time and reducing the chance of staff reaching patients prior to them falling.

Allied health and nurses shared different views on the usefulness of alarms and what allied health's role was and should be with alarms (40). Allied health had active input in some wards, but nil in others. Ward safety huddles were formal opportunities for allied health and nursing to collaboratively create individualised fall prevention plans, including whether patients required/no longer required alarms. Nurses reflected they were often better placed to make alarm decisions due to their presence over 24 h and alarm requirements for behavioural, rather than mobility, reasons (41). To some extent, allied health agreed but valued a team approach among nursing and allied health. Overall, nurses perceived they could overrule allied health's alarm decisions (42).

Some nurses questioned allied health's understanding of the impact of alarms on nurses' workloads (43). Allied health did not report checking with nurses about the number of alarms currently used on the ward prior to recommending additional alarms. Occasionally, nurses suspected physiotherapists recommended alarms as a way of discharging their responsibility, with little review of the effectiveness of alarms or consideration of when they should be removed (44). Allied health expressed an opposing view and believed they often led the team in considering the most appropriate fall prevention interventions, including the appropriateness of alarms (45). Physiotherapists conceded they took a cautious approach and recommended alarms if they anticipated other strategies they recommended, such as regular visual checks, were not feasible due to nurse workloads. The day‐to‐day responsibility to keep patients safe was overwhelmingly borne by nurses (46).

Nurses expressed frustration when allied health viewed responding to alarms as solely a nursing responsibility. Some allied health countered they answered alarms, but usually only if they were in the immediate vicinity. Nurses felt alarms could be more effective if they were a team responsibility (47). Routing alerts via annunciators was viewed as an effective way to generate team responses; nevertheless, this also had disadvantages of disturbing everyone with false alerts.

Interestingly, particularly on acute wards, allied health rarely attended the researchers' ward visits to discuss the evidence around alarm effectiveness and the planned disinvestment trial. Thus, potentially indicating how allied health saw themselves in relation to alarms – very much on the periphery of how wards used them.

#### 5.6.2 Leaders Creating the Culture

If everyone is not on board and the culture isn't developed, you can have all the alarms you want, you can have all the strategies you want, but if you haven't developed the culture of risk management and falls prevention and behaviour expectations, it doesn't matter what you have got around you because it isn't going to change (G‐K.I)

Fall prevention leads and Nurse Unit Managers emphasised the importance of establishing a ward culture that prioritised falls prevention. Leaders felt staff needed reminding to work as a multidisciplinary team for falls prevention, even though this is the essence of comprehensive person‐centred care and considered something staff should do automatically (48).

The roles of Falls Champions and Behaviour of Concern clinicians were rarely raised. Ward‐level nursing leadership was important in allocating workloads, so staff did not have too many high falls risk patients and alarms in their allocation. Equally, staff had responsibilities to feedback to the nurse manager when workloads were unmanageable for alarms to be answered promptly. Some fall prevention leads appeared to have direct contact with wards and problem‐solved with staff complicated fall risk patients where standard strategies, including alarms, were not working. Others offered more generalised non‐patient‐specific support. All fall managers highlighted the need to listen to staff concerns and reasons for alarm reliance prior to any plans to disinvest in them (49).

Managers highlighted the cost‐saving benefit of organisational scrutiny of alarm expenditure (50). Alarms were generally accessible and externally hired with little, or no, clinical justification required. Nurses often had authority to initiate alarms themselves, whereas Constant Observers required manager approval.

### 5.7 Navigating Ambiguity and Fearing Consequences

#### 5.7.1 Navigating Alarm Expectations

The Falls Subcommittee, we don't endorse the use of falls alarms, and they are not included in the hospital policy … but they are used (G‐K.I)

Staff felt organisational expectations that alarms should be used for high fall risk patients (51), often referring to organisation fall guidelines or risk assessment and management tools which listed alarms as an option. Many staff interpreted this as mandating, rather than suggesting, alarms. One organisation restructured their risk assessment and management form; positioning alarms lower in the list to reflect alarms' less certain evidence to discourage their use as a primary strategy. Decisions to cease alarms, even when constantly false alerting, appeared difficult with staff either not allowed (52) or reluctant to make these decisions themselves. Consequently, alarms were left in place “just in case” leading to high alarm usage (53). Nurses felt obliged to implement alarms based on physiotherapists' high falls risk assessment outcomes, despite the assessing physiotherapists deeming alarms unnecessary (54).

One ward previously attempted alarm disinvestment, but they were re‐introduced because alarms were part of the organisational falls prevention toolkit. When a manager highlighted alarms were removed from their guideline, in‐charge nurses reported being unaware of this and shared it was difficult keeping abreast of constantly changing hospital guidelines. The in‐charge nurses reflected they advised staff to implement alarms based on their incorrect assumption they were stipulated in the guidelines.

Fall managers confirmed they did not overtly encourage or discourage alarm use. They emphasised the importance of using additional strategies and not relying solely on alarms. Many staff highlighted they wanted clearer guidance about when alarms were required, for what type of patients, when they should be removed, who should be involved in alarm decisions, and responding to them. Lack of guidance equated to high use. In contrast, staff were confident in organisational guidance surrounding bilateral bedrails, and they were rarely used.

#### 5.7.2 Fear of Reprisal if Alarm Is Not Used

If the hospital gets sued then we'll get sued, because the hospital will sue us. It could happen (D1)

Participants feared liability if alarms were not implemented and reported little faith their organisations would support them in defending their professional reputation if patients fell (55). Despite doubting alarm effectiveness, some Nurse Unit Managers instructed staff to use them purely to save extra post‐fall scrutiny (56). Fear of liability exacerbated disunity between nurses and allied health (57). Nevertheless, team alarm removal decisions were valued to share liability (58).

Many staff on the intervention wards planned for disinvestment viewed trial participation as an opportunity to embrace innovation and risk. They recognised the limitations of using many alarms (false alerts, faults, noise, distraction, sleep disturbance, costs) and considered the trial a forced review of their falls prevention approach and generated discussions about if/how alarms could be used more effectively and in a person‐centred way. Existing fall assessment tools were felt to limit clinical reasoning and encourage a superficial approach where alarms were implemented as a quick‐fix solution to prevent falls and avoid liability (59, 60).

## 6 Discussion

**FIGURE 2:**
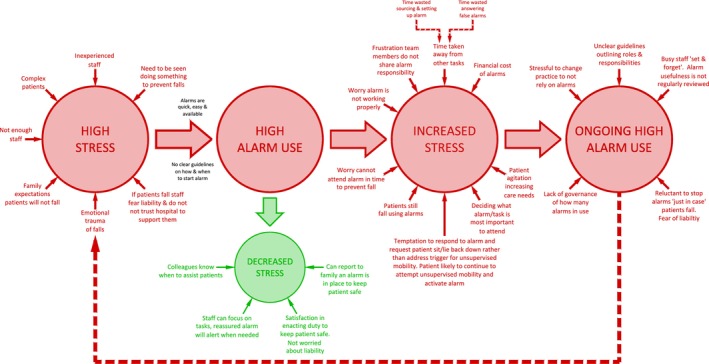
Cycle of stress and alarm use.

This study described why and how staff use alarms in hospitals to prevent falls and suggests part of the reason they are not as effective as hoped is because they are not being used in a person‐centred way. The six themes revealed two overarching paradoxes. First, staff use alarms because they feel under‐resourced, but alarms are less effective because of the resources required to source, set up, and answer alarms. This takes time away from already busy staff. Second, staff use alarms because they fear liability and want to avoid the emotional trauma of a patient falling under their care. However, in practice, alarms add a layer of emotional trauma when staff hear them and cannot respond promptly. Hence, it appears staff factors are the main drivers of alarm use, rather than the more person‐centred drivers of patients' needs, values, and preferences. The misalignment of alarms with person‐centred practice and the cycle of stress perpetuates high alarm use despite their limited effectiveness (see Figure 2). Staff who are stressed and working in cultures where they perceive they will be blamed and criticised if alarms are not used are less likely to be emotionally equipped to engage with patients authentically and holistically.

Alarms were used as a substitute when more frequent person‐centred rounding was not feasible. Intentional rounding is a proactive systematic approach for nurses to check patients at set intervals to address their comfort, pain, and toileting needs, which can reduce falls (Christiansen et al. 2018). However, with high patient complexity, quality of care can be rationed (Mantovan et al. 2020). Participants were fearful that without alarms, patients would fall and lie unnoticed for long periods, increasing their risk of injury severity. Complications arising from long lies post hospital falls could not be found in the literature, though it is noted that patient surveillance is a frequent component of missed care in hospital (Recio‐Saucedo et al. 2018). Alarms fill this surveillance gap but potentially diminish the quality of rounding. Staff might postpone or streamline their rounding interactions, treating them as quickly performed tasks since they know they could respond if alarms alerted them. Under time constraints, staff were tempted to respond to alarms and request patients sit/lie back down rather than take a more person‐centred approach exploring the reason why patients were attempting to mobilise unsupervised. Without mobilisation triggers being addressed, the chances of subsequent alarm activations disrupting staff increased. When alarm alerts increased, so did the risk that alarms were ignored or not responded to promptly. With the infinite needs of complex patient populations but finite staffing resources, rationing of care is unavoidable. However, it is important that alarms are not used at the expense of more person‐centred fall prevention care.

Hospitals are stressful environments for both patients and staff. A meta‐analysis found nurse burnout is associated with increased patient falls (Li et al. 2024). Falls, whether they are major or minor, increase workloads. Like previous research, participants acknowledged the questionable effectiveness and the additional stress alarms created by increasing noise, agitation and wasting time due to false alerts (Considine et al. 2023). Nevertheless, the overwhelming stress falls might occur appear to outweigh the stresses alarms provoke. Our findings concur with Bok et al. (2016) research whereby feeling horrible, guilty, and a sense of failure was experienced by nurses when patients fell and prompted increased alarm use. Nurses have been described as second victims whereby negative feelings of anguish, fear and concern about their professional reputation persist long after the fall (Quadros et al. 2022). Alarms were an attractive option because they could be implemented quickly. This was considered worth it if it meant it could avoid a fall and the stress of post‐fall investigations.

A pillar of PCP is authentic patient involvement in their care. Alarms are predominantly used for patients with cognitive impairment. This poses ethical complexities around partnering with them in their own fall prevention plans due to assumptions they cannot understand or limit their fall risk. Whilst this is the case for some people, research has shown people with cognitive impairment can share insights into their hospital fall prevention and alarm experiences (Stephen and Campbell 2023). Our study lacked examples where patients were involved in alarm decisions in ways that best reflected the importance they assigned to autonomy and safety in hospital. Participants espoused to hold person‐centred values, but at times their behaviour revealed otherwise as alarms were insisted despite an awareness patients disliked them. A systematic review revealed a culture where caring for people with dementia in hospital, a key population who are prescribed alarms, was considered mundane and unrewarding (Houghton et al. 2016). The review revealed staff were kind but not equipped with sufficient skills to work with patients with cognitive impairment. This resulted in de‐personalised care, so safety was met over dignity needs and relational aspects of care were limited due to time constraints.

Residential care settings are perhaps ahead of hospitals in how they involve care recipients and their families in dignity of risk decisions involving alarms. Research has demonstrated falls can be effectively managed by instilling a culture where alarms are used in a considered way, individualised to the resident's behaviour with agreements recorded about residents and families accepting the trade‐off between safety and freedom of movement and privacy (Vandenberg et al. 2017). Whilst some examples were provided in our study where patients were involved in choosing fall prevention options that best reflected their values, there appeared to be a lack of any formal processes to ensure this person‐centred approach was prioritised in the fast‐paced hospital setting.

Families are an important component in PCP. They help patients feel psychologically safe and are a valuable resource given their intimate knowledge of their family member's routines and triggers that might exacerbate confusion (Houghton et al. 2016). Our participants described alarms were not required whilst family were visiting. It is important staff know what level of involvement families wish to have and not impose their own values and assumptions on what they think families ought to be doing to support patients in hospital. Whilst family involvement in care processes is a cultural norm and responsibility in many Asian countries, Western countries do not have this expectation of “hands on” support (Park et al. 2022). What discipline or staff member ‘owned’ the responsibility of engaging families in fall prevention, including decisions to use or not use alarms, was unclear in our study. Hospital fall prevention initiatives recognise the value of family input. An RCT found allied health assistant‐led patient falls education, developed to address workforce shortages and a lack of ownership, reduced falls. However, patients with moderate–severe cognitive impairment were excluded, and those with mild cognitive impairment struggled to engage (Thwaites et al. 2025). The researchers suggested future studies define a clearer role for families to support allied health assistants to deliver education for those with cognitive impairment. It must be noted that whilst family input is encouraged, our participants described instances of verbal abuse by families if an alarm was not used. Unfortunately, occupational violence in hospitals is increasing, with staff a target for family to vent their anger and frustration (Lim et al. 2022). It is unsurprising staff provide alarms (even if they doubt their effectiveness) to appease families and avoid potential abuse.

Good teamwork is another vital component in PCP. Our study highlights the overwhelming view alarms were more effective if using a team approach whereby both decisions to implement, remove and respond to alarms, were shared among all clinicians, rather than just a nursing responsibility. Occasionally, an “us versus them” mindset existed between nurses and allied health, which has been echoed in previous research examining the context of fall prevention in National Health Service hospitals in England (Randell et al. 2024). ‘Not my patient, not my problem’ is an example of a siloed mentality borne out of the need for efficiency and is contrary to a PCP approach which can ultimately save time and be an efficient way to provide care (Australian Commission on safety and quality in health care n.d.). Siloed mentality is defined by not being aware of what others are doing, stuckness, isolation, powerlessness and a lack of trust, respect, collegiality and collaboration (Lau et al. 2024). These characteristics were evident in nurse participants' descriptions of their heightened stress, hopelessness and frustration at their inability to optimally manage many alarms due to high patient complexity, low staffing and lack of assistance of others to respond. However, at the same time they felt stuck, perceiving no other way of managing fall risk other than continued overreliance on alarms. Organisational silos were evident as participants expressed frustration at colleagues in other disciplines that alarms were or were not being used. Recent research demonstrated that while falls prevention is a priority, it is often neglected as staff felt disempowered to address falls risk due to complexities, competing priorities, hierarchical tensions, and workforce stressors. Shared responsibility for falls prevention was identified as a potential solution (McLennan et al. 2024). Sharing responsibility of fall prevention plans across disciplines has also been identified as an opportunity to reduce perceived liability provided there is clarity among multidisciplinary roles and responsibilities (Singh et al. 2022). This enables staff to continue to feel valued and maintain their identity within the team, but with the patient as the focus, rather than themselves.

Leadership is critical in creating a culture of teamwork. Fall prevention managers in our study often had organisational assurance and direct ward supportive elements in their roles. However, it was the Nurse Unit Managers that had ultimate responsibility for ensuring falls risk was assessed and managed appropriately, including overseeing alarm use. Interestingly, most participants in our study were unable to identify a ward‐based ‘falls champion’ to seek guidance from. This could have contributed to high alarm use when it was not appropriate or indeed harmful. Consistent with previous research, our participants described challenges where falls champion positions were vacant, roles unclear, and duties not prioritised due to being too busy in their clinical roles (Randell et al. 2024). Falls champions inspire cohesive staff efforts to improve their fall prevention practices to be consistent with current evidence (Wilson et al. 2016). Many of our participants were grateful the researchers were exploring alarm effectiveness as they knew their current way of using alarms was flawed. Again, residential aged care has been quicker to embrace evidence of alarm ineffectiveness. They found engaging local‐level leadership was critical in successfully sustaining an alarm elimination programme (Hartmann et al. 2021).

A lack of formal alarm procedures and staff awareness of them, and leaders who neither explicitly encouraged nor discouraged the use of alarms meant who, how, and when alarms were implemented or removed was unclear. Combined with a fear of liability, this often led to the over‐prescription of alarms and reluctance to remove them. A systematic review of hospital falls guidelines identified high‐quality guidelines exist and alarms were not recommended in guidelines since 2011 (McKercher et al. 2024). The issue is ensuring staff are aware of, and their workload allows implementation of the guidelines. Hospitals in our study varied in whether alarms were still included in their guidelines or toolkits and whether staff were aware of this detail. Similar findings of staff unfamiliarity with guidelines and defaulting to precautionary approaches were found by Singh et al. (2022). Our participants suggested their organisations' risk‐averse culture drove high alarm use. When managers have an intense focus on goals of zero falls, nurses can feel blame and shame when patients fall and become nervous caring for high fall risk patients, so they resort to alarms and restricting patients' mobility, whereas wards that do not experience this intense messaging encourage mobility (King et al. 2018). Previous research also supports our finding that staff implement alarms despite doubting their effectiveness due to fear of discipline for not strictly adhering to procedures, and the authors recommended managers carefully consider how fall prevention policies are presented (Fehlberg et al. 2020).

### 6.1 Strengths and Limitations

A strength of this study is the exclusive focus on staff attitudes towards alarms. Previously, attitudes towards alarms were researched as a smaller subset within broader falls prevention. The large multi‐site sample included a diverse range of participants across hospitals' acute and subacute wards, disciplines, managers, and frontline staff in public and private sectors in metropolitan and regional areas. To our knowledge, the only other dedicated qualitative research on staff attitudes towards alarms was much smaller on a single ward exclusively with nurses (Considine et al. 2023). We do not claim reaching data saturation given the pragmatic predetermined number of wards and managers interviewed as part of the overarching trials. Nevertheless, given our large sample size and application of the PCPF to the entire dataset, we are confident we have interpreted most aspects of staff's attitudes towards alarms using a person‐centred lens.

This study has several limitations. Including observations beyond the interviews could have strengthened analysis by permitting the researchers to clarify if participants engaged in alarm practices that were not consistent with what they described. Aligning interview questions to the PCPF could have facilitated deeper insights into participants' self‐assessment of how person‐centred their current alarm practice was. However, suboptimal person‐centred practices emerged in the data analysis stage (post data collection). Gathering demographic information on participants' years of experience could have been beneficial as attitudes between experienced and less experienced staff appeared to differ according to whether they had ever worked without alarms as an option. Finally, there may be differential findings if we had focussed discussions specifically on chair versus bed alarms.

### 6.2 Recommendations for Further Research

Research is required to explore how staff undertake disinvesting from high numbers of alarms when they are embedded as routine practice. What is and is not helpful? Does reducing the overuse of alarms result in improved person‐centred fall prevention care? Additionally, advancing technology means alarming devices continue to develop. It is important that both their effectiveness, acceptability, and implications for person‐centred practice are researched from a patient, family, and staff perspective prior to widespread hospital use.

### 6.3 Implications for Policy and Practice

Understanding staff attitudes towards alarms helps to explain why alarms continue to be relied upon in high numbers despite evidence generated a decade ago that indicated they did not significantly reduce falls. Without this contextual knowledge, attempts to reduce alarms will be difficult and high alarm rates are likely to continue and consume much‐needed resources that can be allocated elsewhere.

Adopting a more person‐centred falls prevention approach may reduce overall alarm use or promote more individualised application of alarms. A focus on developed staff interpersonal skills will enable staff to truly listen to patients or observe and respond to nonverbal cues to know when alarms might be causing more harm than good. Nurses can prioritise self‐reflection and remain aware of their stress levels and how they may impact their decisions and responses to alarms. Allied health should avoid recommending alarms from an isolated individual patient perspective but consider how many alarms are currently in use on the ward. Multidisciplinary teams can engage in productive discussions to rationalise the number of alarms and evaluate their usefulness from a ward perspective. Staff should prioritise communication with family to know how to individualise fall prevention plans beyond the application of alarms when patients have cognitive impairment. Whilst it is important to partner with families, staff need to be mindful of the incongruences that can exist between people with cognitive impairment and their family on the importance placed on care values such as autonomy, burden and safety (Miller et al. 2018). Given all these values are potentially impacted by alarms, it is important staff check families have adequate knowledge of the patient's values to act in accordance with them, rather than reactively providing alarms at family members' demands.

Hospital leaders could consider removing alarms as an option on management plans linked to fall risk assessment tools given staff's tendency to interpret this as a mandatory strategy. Fall prevention guidelines should outline staff responsibilities while ensuring adequate autonomy for staff to use their own judgement to determine risk versus benefit when deciding on fall prevention interventions. Finally, leaders should foster a culture that emphasises steadfast organisational support, rather than apportioning blame.

## 7 Conclusion

Staff feel compelled to use alarms despite the multitude of problems associated with their use and challenges to person‐centred practice. The more alarms in use, the less likely they were individualised to patients' needs and preferences. Drivers of alarm use were feeling under‐resourced and fearing liability if a patient fell. A discordance between nursing and allied health existed in the value placed on alarms and how fall prevention was approached as a team. Staff want clearer organisational guidance in the use of alarms, but at the same time want the freedom to use their own clinical reasoning. Alarm disinvestment and the extent to which it can be implemented warrant further investigation.

## Author Contributions

All authors made substantial contributions to conception and design, or acquisition of data, or analysis and interpretation of data: K.S., D.P., J.W.‐N. and T.P.H.; Involved in drafting the manuscript or revising it critically for important intellectual content: K.S., D.P., J.W.‐N. and T.P.H.; Given final approval of the version to be published. Each author should have participated sufficiently in the work to take public responsibility for appropriate portions of the content: K.S., D.P., J.W.‐N. and T.P.H.; Agreed to be accountable for all aspects of the work in ensuring that questions related to the accuracy or integrity of any part of the work are appropriately investigated and resolved: K.S., D.P., J.W.‐N. and T.P.H.

## Conflicts of Interest

K.S. is an employee of Eastern Health where some participants were recruited but does not work at the specific hospital of recruitment. T.P.H. has provided Expert Witness Testimony on the subject of falls prevention in hospital for MinterEllison Law Firm in the past three years, with payment directed to his institution (Monash University).

## Supporting information


**Data S1:** Supporting Information

**Data S2:** Supporting Information

## Data Availability

The data that support the findings of this study are available from the corresponding author upon reasonable request.

## References

[jan70113-bib-0001] Australian Commission on safety and quality in health care . n.d. “Rationale and Approaches to Person‐Centred Care.” https://www.safetyandquality.gov.au/our‐work/partnering‐consumers/person‐centred‐care/rationale‐and‐approaches‐person‐centred‐care.

[jan70113-bib-0002] Barker, A. L. , R. T. Morello , D. R. Ayton , et al. 2017. “Acceptability of the 6‐PACK Falls Prevention Program: A Pre‐Implementation Study in Hospitals Participating in a Cluster Randomized Controlled Trial.” PLoS One 12, no. 2: e0172005. 10.1371/journal.pone.0172005. 28199376 PMC5310900

[jan70113-bib-0003] Bok, A. , L. L. Pierce , C. Gies , and V. Steiner . 2016. “Meanings of Falls and Prevention of Falls According to Rehabilitation Nurses: A Qualitative Descriptive Study.” Rehabilitation Nursing 41, no. 1: 45–53. 10.1002/rnj.221. 26332851

[jan70113-bib-0004] Brusco, N. K. , A. M. Hutchinson , D. Mitchell , et al. 2021. “Mobilisation Alarm Triggers, Response Times and Utilisation Before and After the Introduction of Policy for Alarm Reduction or Elimination: A Descriptive and Comparative Analysis.” International Journal of Nursing Studies 117: 103769. 10.1016/j.ijnurstu.2020.103769. 33647843

[jan70113-bib-0005] Christiansen, A. , L. Coventry , R. Graham , E. Jacob , D. Twigg , and L. Whitehead . 2018. “Intentional Rounding in Acute Adult Healthcare Settings: A Systematic Mixed‐Method Review.” Journal of Clinical Nursing 27, no. 9–10: 1759–1792. 10.1111/jocn.14370. 29603820

[jan70113-bib-0006] Considine, J. , D. Berry , M. Mullen , et al. 2023. “Nurses' Experiences of Using Falls Alarms in Subacute Care: A Qualitative Study.” PLoS One 18, no. 6: e0287537. 10.1371/journal.pone.0287537. 37347774 PMC10286966

[jan70113-bib-0007] Dykes, P. C. , M. Curtin‐Bowen , S. Lipsitz , et al. 2023. “Cost of Inpatient Falls and Cost‐Benefit Analysis of Implementation of an Evidence‐Based Fall Prevention Program.” JAMA Health Forum 4, no. 1: e225125. 10.1001/jamahealthforum.2022.5125. 36662505 PMC9860521

[jan70113-bib-0008] Fehlberg, E. A. , C. L. Cook , R. I. Bjarnadottir , A. M. McDaniel , R. I. Shorr , and R. J. Lucero . 2020. “Fall Prevention Decision Making of Acute Care Registered Nurses. *JONA* .” Journal of Nursing Administration 50, no. 9: 442–448. 10.1097/nna.0000000000000914. 32826513 PMC7592292

[jan70113-bib-0009] Haines, T. P. , M. Botti , N. Brusco , et al. 2021. “Disinvestment in the Presence of Uncertainty: Description of a Novel, Multi‐Group, Disinvestment Trial Design and Protocol for an Application to Reduce or Cease Use of Mobilisation Alarms for Preventing Falls in Hospitals.” PLoS One 16, no. 12: e0261793. 10.1371/journal.pone.0261793. 34969050 PMC8717976

[jan70113-bib-0010] Hartmann, C. W. , C. Gillespie , G. G. Sayre , and A. L. Snow . 2021. “De‐Implementing and Sustaining an Intervention to Eliminate Nursing Home Resident Bed and Chair Alarms: Interviews on Leadership and Staff Perspectives.” Implementation Science Communications 2, no. 1: 91. 10.1186/s43058-021-00195-w. 34429167 PMC8383405

[jan70113-bib-0011] Houghton, C. , K. Murphy , D. Brooker , and D. Casey . 2016. “Healthcare Staffs' Experiences and Perceptions of Caring for People With Dementia in the Acute Setting: Qualitative Evidence Synthesis.” International Journal of Nursing Studies 61: 104–116. 10.1016/j.ijnurstu.2016.06.001. 27343469

[jan70113-bib-0012] Hubbartt, B. , S. G. Davis , and D. D. Kautz . 2011. “Nurses' Experiences With Bed Exit Alarms May Lead to Ambivalence About Their Effectiveness (CE).” Rehabilitation Nursing 36, no. 5: 196–199. 10.1002/j.2048-7940.2011.tb00195.x. 21882797

[jan70113-bib-0013] Janerka, C. , G. D. Leslie , and F. J. Gill . 2023. “Development of Patient‐Centred Care in Acute Hospital Settings: A Meta‐Narrative Review.” International Journal of Nursing Studies 140: 104465. 10.1016/j.ijnurstu.2023.104465. 36857979

[jan70113-bib-0014] Kerr, L. , P. Newman , and P. Russo . 2023. “‘I Don't Want to Impose on Anybody’: Older People and Their Families Discuss Their Perceptions of Risk, Cause and Care in the Context of Falls.” International Journal of Older People Nursing 18: e12578. 10.1111/opn.12578. 37776081

[jan70113-bib-0015] King, B. , K. Pecanac , A. Krupp , D. Liebzeit , and J. Mahoney . 2018. “Impact of Fall Prevention on Nurses and Care of Fall Risk Patients.” Gerontologist 58, no. 2: 331–340. 10.1093/geront/gnw156. 28011591 PMC5946811

[jan70113-bib-0016] Lau, R. S. , M. E. Boesen , L. Richer , and M. D. Hill . 2024. “Siloed Mentality, Health System Suboptimization and the Healthcare Symphony: A Canadian Perspective.” Health Research Policy and Systems 22, no. 1: 87. 10.1186/s12961-024-01168-w. 39020412 PMC11253392

[jan70113-bib-0017] Li, L. Z. , P. Yang , S. J. Singer , J. Pfeffer , M. B. Mathur , and T. Shanafelt . 2024. “Nurse Burnout and Patient Safety, Satisfaction, and Quality of Care.” JAMA Network Open 7, no. 11: e2443059. 10.1001/jamanetworkopen.2024.43059. 39499515 PMC11539016

[jan70113-bib-0018] Lim, M. C. , M. S. Jeffree , S. S. Saupin , N. Giloi , and K. A. Lukman . 2022. “Workplace Violence in Healthcare Settings: The Risk Factors, Implications and Collaborative Preventive Measures.” Annals of Medicine and Surgery 78: 103727. 10.1016/j.amsu.2022.103727. 35734684 PMC9206999

[jan70113-bib-0019] Lumivero . 2023. “NVivo (Version 14).” www.lumivero.com.

[jan70113-bib-0020] Mantovan, F. , C. Muzzana , M. Schubert , and D. Ausserhofer . 2020. ““It's About How We Do It, Not if We Do It”. Nurses' Experiences With Implicit Rationing of Nursing Care in Acute Care Hospitals: A Descriptive Qualitative Study.” International Journal of Nursing Studies 109: 103688. 10.1016/j.ijnurstu.2020.103688. 32668336

[jan70113-bib-0021] McCance, T. , and B. McCormack . 2021. “The Person‐Centred Practice Framework.” In Fundamentals of Person‐Centred Healthcare Practice, edited by B. McCormack , T. McCance , S. Martin , A. McMillan , and C. Bulley . Wiley.

[jan70113-bib-0022] McKercher, J. P. , C. L. Peiris , A. Hill , et al. 2024. “Hospital Falls Clinical Practice Guidelines: A Global Analysis and Systematic Review.” Age and Ageing 53, no. 7: afae149. 10.1093/ageing/afae149. 39023234 PMC11255989

[jan70113-bib-0023] McLennan, C. , C. Sherrington , W. Tilden , et al. 2024. “Considerations Across Multiple Stakeholder Groups When Implementing Fall Prevention Programs in the Acute Hospital Setting: A Qualitative Study.” Age and Ageing 53, no. 10: afae208. 10.1093/ageing/afae208. 39354814 PMC11445322

[jan70113-bib-0024] Miller, L. M. , C. J. Whitlatch , C. S. Lee , and K. S. Lyons . 2018. “Incongruent Perceptions of the Care Values of Hospitalized Persons With Dementia: A Pilot Study of Patient‐Family Caregiver Dyads.” Aging & Mental Health 22, no. 4: 489–496. 10.1080/13607863.2017.1280766. 28128641 PMC5529266

[jan70113-bib-0025] Mitchell, D. , M. Raymond , J. Jellett , et al. 2018. “Where Are Falls Prevention Resources Allocated by Hospitals and What Do They Cost? A Cross Sectional Survey Using Semi‐Structured Interviews of Key Informants at Six Australian Health Services.” International Journal of Nursing Studies 86: 52–59. 10.1016/j.ijnurstu.2018.06.002. 29966825

[jan70113-bib-0026] Montero‐Odasso, M. , N. van der Velde , F. C. Martin , et al. 2022. “World Guidelines for Falls Prevention and Management for Older Adults: A Global Initiative.” Age and Ageing 51, no. 9: afac205. 10.1093/ageing/afac205. 36178003 PMC9523684

[jan70113-bib-0027] Morris, M. E. , K. Webster , C. Jones , et al. 2022. “Interventions to Reduce Falls in Hospitals: A Systematic Review and Meta‐Analysis.” Age and Ageing 51, no. 5: afac077. 10.1093/ageing/afac077. 35524748 PMC9078046

[jan70113-bib-0028] Okumoto, A. , C. Miyata , S. Yoneyama , and A. Kinoshita . 2020. “Nurses' Perception of the Bed Alarm System in Acute‐Care Hospitals.” SAGE Open Nursing 6: 2377960820916252. 10.1177/2377960820916252. 33415274 PMC7774491

[jan70113-bib-0029] Park, J. Y. , J. F. Pardosi , M. S. Islam , T. Respati , K. Chowdhury , and H. Seale . 2022. “What Does Family Involvement in Care Provision Look Like Across Hospital Settings in Bangladesh, Indonesia, and South Korea?” BMC Health Services Research 22, no. 1: 922. 10.1186/s12913-022-08278-7. 35841023 PMC9286761

[jan70113-bib-0030] Prato, L. , L. Lindley , M. Boyles , L. Robinson , and C. Abley . 2019. “Empowerment, Environment and Person‐Centred Care: A Qualitative Study Exploring the Hospital Experience for Adults With Cognitive Impairment.” Dementia 18, no. 7–8: 2710–2730. 10.1177/1471301218755878. 29411662

[jan70113-bib-0031] Pu, D. , D. Mitchell , N. Brusco , et al. 2024. “Does the Site of Research Evidence Generation Impact on Its Translation to Clinical Practice? A Protocol Paper.” PLoS One 19, no. 12: e0314956. 10.1371/journal.pone.0314956. 39671381 PMC11643258

[jan70113-bib-0032] Quadros, D. V. , A. M. Magalhães , P. Wachs , I. M. Severo , J. P. Tavares , and D. Dal Pai . 2022. “Modeling of Adult Patient Falls and the Repercussions to Nursing as a Second Victim.” Revista Latino‐Americana de Enfermagem 30: e3618. 10.1590/1518-8345.5830.3618. PMC9342907 35920541

[jan70113-bib-0033] Randell, R. , L. McVey , J. Wright , et al. 2024. “Practices of Falls Risk Assessment and Prevention in Acute Hospital Settings: A Realist Investigation.” Health and Social Care Delivery Research 12: 1–194. 10.3310/jwqc5771. 38511977

[jan70113-bib-0034] Recio‐Saucedo, A. , C. Dall'Ora , A. Maruotti , et al. 2018. “What Impact Does Nursing Care Left Undone Have on Patient Outcomes? Review of the Literature.” Journal of Clinical Nursing 27, no. 11–12: 2248–2259. 10.1111/jocn.14058. 28859254 PMC6001747

[jan70113-bib-0035] Ritchie, J. , L. Spencer , and W. O'Conner . 2003. “Carrying out Qualitative Analysis.” In Qualitative Research Practice: A Guide for Social Science Students and Researchers. SAGE.

[jan70113-bib-0036] Singh, H. , K. Collins , H. M. Flett , S. B. Jaglal , and K. E. Musselman . 2022. “Therapists' Perspectives on Fall Prevention in Spinal Cord Injury Rehabilitation: A Qualitative Study.” Disability and Rehabilitation 44, no. 16: 4351–4360. 10.1080/09638288.2021.1904013. 33789064

[jan70113-bib-0037] Stephen, K. , and A. Campbell . 2023. “The Experiences of Older Adults With Cognitive Impairment in Using Falls Prevention Alarms in Hospital: A Qualitative Descriptive Study.” Australian Occupational Therapy Journal 71, no. 1: 132–148. 10.1111/1440-1630.12917. 38016634

[jan70113-bib-0038] Thorne, S. 2016. Interpretive Description: Qualitative Research for Applied Practice. 2nd ed. Routledge.

[jan70113-bib-0039] Thwaites, C. , L. Shaw , R. Lui , et al. 2025. “Boosting Hospital Falls Prevention Using Health Assistant Staff Alongside Usual Care.” Patient Education and Counseling 130: 108464. 10.1016/j.pec.2024.108464. 39418674

[jan70113-bib-0040] Vandenberg, A. E. , B. Van Beijnum , V. G. Overdevest , E. Capezuti , and T. M. Johnson . 2017. “US and Dutch Nurse Experiences With Fall Prevention Technology Within Nursing Home Environment and Workflow: A Qualitative Study.” Geriatric Nursing 38, no. 4: 276–282. 10.1016/j.gerinurse.2016.11.005. 27956058 PMC6546295

[jan70113-bib-0041] Wilson, D. S. , M. Montie , P. Conlon , M. Reynolds , R. Ripley , and M. G. Titler . 2016. “Nurses' Perceptions of Implementing Fall Prevention Interventions to Mitigate Patient‐Specific Fall Risk Factors.” Western Journal of Nursing Research 38, no. 8: 1012–1034. 10.1177/0193945916644995. 27106881

