# Supplementary material for: Mobilisation Alarm Use in Hospitals and Alignment With Person‐Centred Practice: A Qualitative Study
Source: J Adv Nurs. 2025 Aug 4;82(4):3489–508. doi: 10.1111/jan.70113 (PMC12994649; doi:10.1111/jan.70113)
Supplement: Supplementary file 2 — Data S2: Supporting Information [file JAN-82-3489-s001.docx]

**Supplementary file 2: Semi structured interview guides:** **How and why staff use alarms in hospitals**

**Time point 1: Baseline**

** Majority of data for this study came from baseline interviews*

| **Reaction to workshop / evidence presented / disinvestment study to be undertaken** | |
| --- | --- |
| 1 | How do you feel about the evidence that was just presented regarding the effectiveness of mobilisation alarms in preventing falls? |
| 2 | Clinicians and managers often have a different personal perception about what works based on what they have observed to work in their own setting. Please rank the following 5 falls prevention strategies in terms of what you personally think works, from the least to the most, in your own setting:   \| 1 = Least effective 5 = Most effective \|  \| \| --- \| --- \| \| Non-slip socks \|  \| \| Individualised patient education with a health professional \|  \| \| Mobilisation alarms \|  \| \| Constant patient observers / companions (paid) \|  \| \| Falls risk alert signs for high risk patients \|  \|  - *How did you decide to rank them in this way?* - *Why did you rank # as more / less effective than # in your setting?* - *What advantages / disadvantages do alarms have compared to other falls prevention strategies?* - *Do you think that any of these options are not effective at all?* - *Do you think that any of these options are potentially harmful?* |
| 3 | How do you feel about the removal of mobilisation alarms from your hospital ward for the purpose of research?  How do you feel about the removal of mobilisation alarms from hospital wards as a part of regular clinical practice? |
| 4 | What involvement, if any, did you have in your ward deciding to be a part of this research project? |
| 5 | How does this project compare with other existing falls prevention projects that your ward may be involved in? |
| **Description of current alarm practices / processes** | |
| 6 | How do you decide if a patient requires a mobilisation alarm? |
| 7 | Can you discuss your ability to respond and provide the required assistance when mobilisation alarms are triggered? |
| 8 | Have you been trained in the optimal set-up of mobilisation alarms (i.e. positioning of device, setting of parameters?) |
| 9 | Does anything trigger the decision to remove a patient’s mobilisation alarm? |
| **Specific fall manager questions** | |
| 1 | Have you had any thoughts or discussions about using mobilisation alarms in the past 12 months? |
| 2 | How are mobilisation alarms distributed and monitored throughout your organisation? What is your role in guiding staff in their clinical reasoning? |
| **Idealised perceptions of mobilisation alarms** | |
| 11 | In your opinion, is there an ideal amount of patients using mobilisation alarms at any one time on a ward? |
| 12 | In your opinion, for mobilisation alarms to be more effective in preventing falls in hospital, what do you think has to happen? |
| **Is there anything else that you would like to share about your thoughts or feelings regarding the effectiveness of mobilisation alarms, the disinvestment study taking place or the way mobilisation alarms are used in hospitals?** | |

** Majority of data generated from time point 1 and 2 will be explored in a subsequent paper exploring staff’s experiences in disinvestment*

**Time point 2: Approximately one week prior to wards transitioning to active phase of reducing/eliminating alarms**

| 1 | What do you think about the use of alarms in falls prevention on your ward? |
| --- | --- |
| 2 | Has there been a change in the way alarms are perceived since baseline workshop?  - Are attitudes different now that removal / elimination of alarms is imminent?  - Contrast with site’s baseline response …e.g “Last time we were here most staff felt … Has this changed?” |
| 3 | Has there been a change in the way alarms are used since baseline workshop?  - What is the change?  - How did it happen?  - Who was involved?  - Patient / family / staff response to change? |
| 4 | Is there a plan as to how you are going to implement the eliminated / reduced alarm condition?  - Who has been involved in these discussions? (i.e. solely nursing, allied health, medical, management).  - Formal Vs informal discussions.  - Any key influential person (champion) that will lead / drive success of the ward’s participation?  - Are you confident that the ward can reduce / eliminate alarms?  - Do you foresee any barriers? How will these be managed?  - What do you hope to see at your ward level as a result of the change in use of alarms? |

**Time point 3: Post intervention**

| **Intervention arm – ward staff** | |
| --- | --- |
| 1 | What are your overall impressions of the evidence we just presented? |
| 2 | What does this evidence mean for your clinical practice? |
| 3 | How did you find participating in this trial?  Did participating in this trial make you look at other areas of your practice differently? |
| 4 | What guidance would you like to see from management regarding making decisions whether to use alarms and how much on the wards? |
| **External impact arm – ward staff** | |
| 1 | What are your overall impressions of the evidence we just presented? |
| 2 | What does this evidence mean for your clinical practice? |
| 3 | Have you made any changes to the way you use alarms in the 12 months since our last visit? What are these changes? |
| 4 | What guidance would you like to see from management regarding making decisions whether to use alarms and how much on the wards? |
| **Key informant** | |
| 1 | What are your overall impressions of the evidence we just presented? |
| 2 | How important is it to you to make a clear decision about the use of alarms or not, compared to other decisions you need to make for the organisation at the moment? |
| 3 | Beside the evidence presented today, what other factors (financial, staff/patient experience) might influence your decision to use alarms? |
| 4 | Since the trial commenced at this organisation, can you share with us any discussions that you may have had about the use of alarms? Who have you had these discussions with? |
| 5 | Do you anticipate your organisation will make certain recommendations regarding alarm use:   - What type of changes in alarm use might be recommended? - Who would lead the implementation of any proposed changes? - How do you anticipate any proposed changes will be embraced by staff? Facilitators / barriers? - How would you plan to roll out any proposed changes? - How would you communicate the proposed changes to frontline staff? - What sort of resources or support do you think staff will need to adapt to the proposed changes? - How would the impact of these changes be reviewed / monitored? What will be captured (falls, alarm use, staff / pt experience)? |
| **Key informant - Intervention arm** | |
| 1 | How do you think your ward/s experienced the reduced/eliminated condition? |
| 2 | Did you have any direct involvement when the wards transitioned? |
| 3 | Can you describe any positive or negative outcomes from participating in the trial? |
| 4 | Can you describe any unanticipated challenges or benefits? |
| 5 | Are you aware if the ward’s use of alarms has changed since completion of the trial? How? |
